# Clinical and Functional Assays of Radiosensitivity and Radiation-Induced Second Cancer

**DOI:** 10.3390/cancers9110147

**Published:** 2017-10-27

**Authors:** Mohammad Habash, Luis C. Bohorquez, Elizabeth Kyriakou, Tomas Kron, Olga A. Martin, Benjamin J. Blyth

**Affiliations:** 1 Cancer Research Division, Peter MacCallum Cancer Centre, 305 Grattan Street, Parkville, VIC 3000, Australia; mohammed.habash@petermac.org (M.H.); benjamin.blyth@petermac.org (B.J.B.); 2 Faculty of Medicine, Dentistry & Health Sciences, The University of Melbourne, Parkville, VIC 3010, Australia; 3 Physical Sciences, Peter MacCallum Cancer Centre, 305 Grattan Street, Parkville, VIC 3000, Australia; carlos.bohorquez@petermac.org (L.C.B.); elizabeth.kyriakou@petermac.org (E.K.); tomas.kron@petermac.org (T.K.); 4 Radiation Oncology and Cancer Imaging, Peter MacCallum Cancer Centre, 305 Grattan Street, Parkville, VIC 3000, Australia; 5 The Sir Peter MacCallum Department of Oncology, The University of Melbourne, Parkville, VIC 3010, Australia

**Keywords:** radiosensitivity, second cancer, radiotherapy, radiogenomics, functional assay

## Abstract

Whilst the near instantaneous physical interaction of radiation energy with living cells leaves little opportunity for inter-individual variation in the initial yield of DNA damage, all the downstream processes in how damage is recognized, repaired or resolved and therefore the ultimate fate of cells can vary across the population. In the clinic, this variability is observed most readily as rare extreme sensitivity to radiotherapy with acute and late tissue toxic reactions. Though some radiosensitivity can be anticipated in individuals with known genetic predispositions manifest through recognizable phenotypes and clinical presentations, others exhibit unexpected radiosensitivity which nevertheless has an underlying genetic cause. Currently, functional assays for cellular radiosensitivity represent a strategy to identify patients with potential radiosensitivity before radiotherapy begins, without needing to discover or evaluate the impact of the precise genetic determinants. Yet, some of the genes responsible for extreme radiosensitivity would also be expected to confer susceptibility to radiation-induced cancer, which can be considered another late adverse event associated with radiotherapy. Here, the utility of functional assays of radiosensitivity for identifying individuals susceptible to radiotherapy-induced second cancer is discussed, considering both the common mechanisms and important differences between stochastic radiation carcinogenesis and the range of deterministic acute and late toxic effects of radiotherapy.

## 1. Introduction

The well-characterized direct and indirect effects of radiation on tumor cell DNA underlie the effectiveness of radiotherapy in killing cancer cells. The ability to deliver high doses in a very conformal fashion to a target has improved significantly over the last years. However, it has proven challenging to fully understand the effects of radiotherapy on surviving tumor cells, on healthy and cancer-associated tissues in the irradiated volume, in low dose irradiated or unirradiated distant tissues, as well as systemic effects in radiotherapy patients. The adverse effects associated with radiotherapy include the risk of a radiation-induced second cancer, with epidemiological evidence suggesting that around 8% of secondary solid tumors can be attributed to radiotherapy [1,2], though this estimate varies by cancer type. Exact figures are confounded by the various patient- and treatment-specific factors that also influence second cancer risk. These include age, gender, lifestyle factors, primary cancer type, other treatments including chemotherapy, radiotherapy modalities and dosimetry [3,4]. However, the largest contributor to variability in adverse events is genetic predisposition [5]. In parallel with efforts to reduce the radiation dose received by healthy tissues during radiotherapy, the evaluation and prediction of radiosensitivity is one of the major opportunities to mitigate the risks of radiotherapy-induced second cancer.

In addition to the extreme radiosensitivity of rare individuals who also display clinical manifestations of inherited mutations, including associated syndromic neoplasms, the accumulation of genomic, epigenetic and other phenotypic data on radiotherapy patients who exhibit unexpected radiosensitivity is beginning to reveal the more subtle degrees of radiosensitivity which define the general population. This emerging field of radiogenomics, characterized by systems biology approaches paired with clinical and epidemiology investigations [6], has begun to undercover the genetic determinants of radiosensitivity, while also producing new questions. Here, current research in radiogenomics, radiosensitivity and their impact on radiation-induced second cancers will be reviewed, with a focus on practical issues facing integration of functional assays of radiosensitivity into clinical treatment planning.

## 2. Observed Susceptibility to Radiation-Induced Toxicity

**Figure 1:**
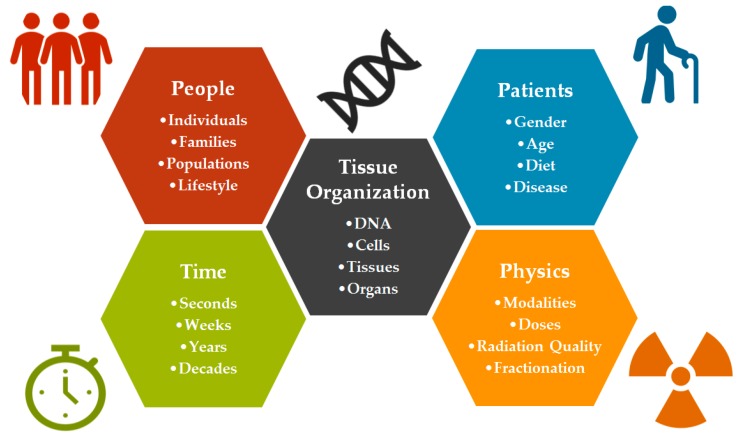
The multi-dimensional problem of radiosensitivity. Radiosensitivity exists across multiple scales of people and time, contributing to the difficulties in predicting who will be sensitive to toxic and carcinogenic effects of radiotherapy in advance.

**Figure 2:**
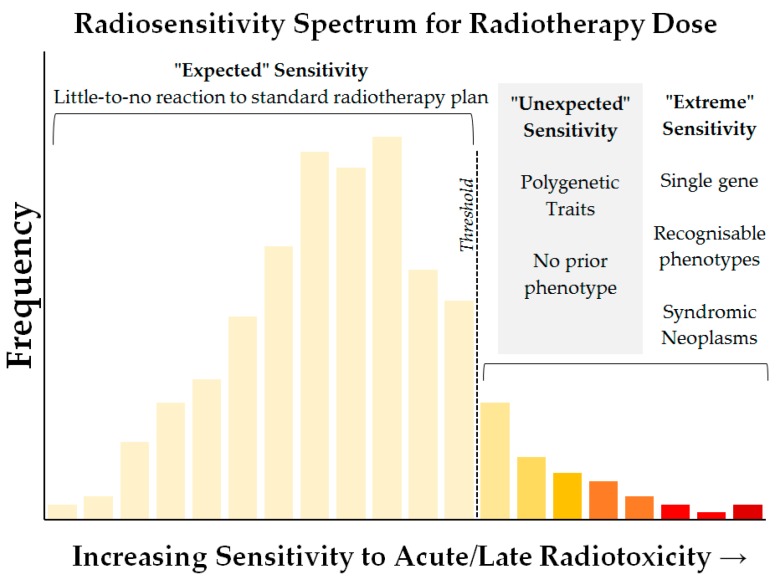
The concept of a radiosensitivity spectrum. Like other polygenetic traits, the true degree of radiosensitivity is expected to cover a wide spectrum rather than a clear bimodal distribution of sensitive and insensitive individuals, even though a binary classification may be apparent for a given clinical endpoint. Given that most acute/late reactions to radiotherapy show threshold responses and these are used to guide dose planning and prescription, sensitivities below the effective threshold for a given clinical toxicity endpoint would be expected to show little-to-no reaction. The patients that do show significant reactions would include those which show extreme sensitivity that might be caused by an inherited single-gene trait, and which might show a recognizable phenotype that might warn of an extreme response. The remaining reactions occur in individuals with no prior phenotype, whose sensitivity was unexpected and is likely a complex polygenetic trait. A singular spectrum of generic radiosensitivity is only an illustrative concept, given the divergent and bimodal expressions of different toxic reactions observed in the clinic, but at the cellular level it can be demonstrated using functional assays and for particular endpoints with careful assessment of reaction grades [7].

Radiosensitivity is a multi-dimensional problem (Figure 1) that spans levels of tissue organization (DNA, cells, tissues), time (from seconds to decades), people (individuals, families and populations), patients (gender, age, health and disease), and physics (modalities, doses and radiation quality) and describes a spectrum of outcomes that are mediated by sometimes competing mechanisms. The name itself can be misleading, given that all radiotherapy patients are expected to experience damage in irradiated tissues such that there are no true radiation-insensitive individuals. Rather, the term describes the distribution of patients along a spectrum of sensitivity (Figure 2) from less-than-expected to more-than-expected [7]. Yet, the fact that there is an expected tolerance to radiation exposure demonstrates that the spectrum is defined by a majority of patients who show no or mild adverse effects when given a standardized treatment course. In fact, such prescriptions have largely been determined by the overall tolerance of the patient population to a given radiation regimen, to avoid acute toxicity which prevents the completion of treatment. This observed tolerance is the result of effective dose thresholds which characterize the various acute and late effects of radiation exposure to healthy tissues.

When radiation doses are limited to levels that minimize cell death in normal tissues, prevent excessive inflammation and preserve the population of stem cells, irradiated normal tissues are resistant to permanent damage and will not show clinically-relevant adverse effects. Yet in radiosensitive individuals, the same radiation dose can induce adverse effects ranging from moderate to severe. Acute effects that occur in proliferating tissues include erythema, dermatitis, hair loss, diarrhea and cystitis. Late effects include fibrosis, atrophy, vascular damage, hormone deficiencies, and infertility (reviewed in [8]). Radiosensitive individuals may show an enhanced susceptibility to one or more reaction, or may exhibit a general sensitivity to various effects. The adverse effects in radiosensitive individuals are generally similar in nature and severity to those that could be observed in the general population at much higher doses, rather than representing unique or novel injuries. It is these altered thresholds for the deterministic effects of radiation that characterize clinical radiosensitivity. Although an individual’s sensitivity can change with age and health status, genetic factors are thought to account for most of the observed variation in radiation sensitivity.

## 3. Observed Susceptibility to Radiation-Induced Cancer

With radiation-induced second malignancies often considered an additional late adverse effect of radiotherapy, the inclusion of this risk in treatment planning and decision-making has been growing, with notable examples of sparing dose to contralateral breast [9,10] and hesitation to use intensity modulated radiotherapy (IMRT) in pediatric cases to avoid exposure to leakage dose [11,12]. The stochastic nature of radiation-induced cancer differs from the deterministic effects described above as it is currently accepted that no dose-threshold exists below which cancers are not expected to be induced [13], nor is the severity of any induced cancer determined by the dose or the radiosensitivity of the individual. Rather, it is the probability of inducing a malignancy for a given exposure which differs. Radiotherapy-induced (second) cancer risk is a specific case of the general radiation-induced cancer risk [14] as observed in epidemiology studies of accidentally- or occupationally-exposed populations, or in studies of Japanese atomic bomb survivors [15]. There are significant uncertainties about the shape of the dose–response curve at very low doses (albeit below the doses received in normal tissues during radiotherapy) [16,17], and likewise competing effects between mutation-induction and cell-killing at very high doses; yet, direct observation of second cancer rates in radiotherapy patients likely incorporates these various sources of uncertainty into the attributable fraction estimates [1,2].

**Figure 3:**
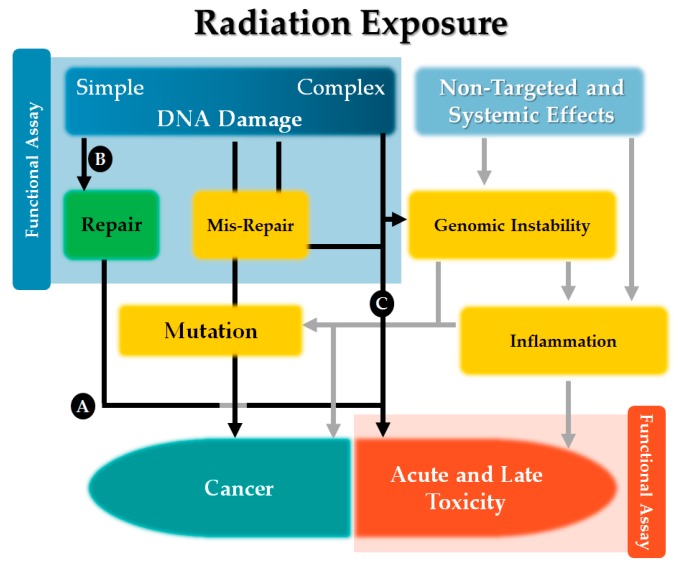
Mechanisms of cancer and tissue toxicity induced by radiation. Radiation primarily induces both cancer and tissue toxicity through its induction of simple and complex DNA damage in individual cells. Accurate repair, misrepair or failure to repair this DNA damage determines the fate of each cell. The fixation of DNA mutations increases the risk of malignant transformation, while irreparable DNA damage should be lethal. The effects of different genes which may contribute to radiosensitivity (Gene A, B or C) may interfere with the functioning of various parts of the pathway, and might alter the likelihood of cell death, or transformation, or both outcomes. Carriers of a particular genetic trait (Gene A) might be sensitive to acute toxicity due to increased levels of cell death even in cells that were appropriately repaired, but this would likely not increase sensitivity to cancer. Although understanding the roles of relevant genes in these pathways improves the predictive power of genomic data, the contribution of non-targeted and systemic effects and interactions between pathways and interactions between multiple genetic traits makes functional assays a useful surrogate measure of phenotype. Yet, a functional assay which only assesses DNA repair might detect the effect of a variant in Gene B, but not a variant Gene C. Likewise, functional assays of apoptosis might be useful surrogates of acute and late toxicity, but might not be informative for second cancer risk.

Whilst the primary mechanism implicated in increased cancer risk in radiation-exposed populations is the induction of mutations following radiation-induced DNA damage leading to an increased number of initiated/pre-malignant cells, it should be recognized that additional mechanisms such as radiation effects on the microenvironment [18,19], altered selection pressures in repopulating tissues [14,20], bystander effects in unirradiated cells and abscopal effects in distant tissues [21], and promotion of pre-existing initiated cells [22] could also mediate radiation effects on cancer incidence. With these mechanisms, the dose to the tumor and the whole-body dose distribution could also be determinants of risk in addition to the discrete dose received by an organ at risk of a second cancer. Just as sensitivity to one deterministic effect of radiation in normal tissues need not be coincident with sensitivity to other manifestations of radiotoxicity [6], it is also reasonable to assume that any altered susceptibility to radiation-induced cancer in the same individual might be highly dependent on the actual pathways which mediate the sensitivity (Figure 3).

The proportion of second malignancies that can be attributed to radiation therapy is based on epidemiological analysis of the rates associated with different treatments [23], coupled with the wider mechanistic understanding of the cancer risks associated with radiation exposure. Such analyses are complicated by several competing causes of second cancers, including: spontaneous co-incident tumors in a given individual (including the various environmental, health and lifestyle factors that underlie general cancer susceptibility); cancers caused by other treatment methods such as chemotherapy [24]; and, second cancers arising in inherently cancer-prone individuals.

Studies of the highly informative Childhood Cancer Survivor Study have revealed key information about the incidence of second cancers and their relationship to treatment, including radiotherapy (reviewed in [25]). The two most frequent second cancers observed in the survivors of childhood cancer are breast and thyroid cancers, with second breast tumors only observed in females, and the majority of thyroid cancers also occurring in females. However, this distribution is consistent with the baseline frequencies and the sex differences observed in the general population for these cancers, rather than an effect of treatment [26]. The effect of age at the time of the primary cancer (and thus the age at radiotherapy) is also complicated by the changing risks with age in the general population [27], with some earlier data supporting increased susceptibility to second breast cancer with primary cancer diagnosis at older ages [28], while later analyses suggest this is the result of increasing incidence of the background rates of breast cancer with age. Other studies show clear increases in the incidence of glioma with both increasing radiation dose and decreasing age at treatment for first cancer [29]. The data suggest that not only does radiation-induced cancer risk change with age, but the spectrum of spontaneous and treatment-associated second malignancies observed, risk factors and latency differ between survivors of childhood and adult-onset primary cancer [30].

The occurrence of second cancers within radiation treatment fields and those that show radiation dose–response relationships provide more specific evidence for radiation-associated second malignancies (reviewed in [31,32]). The most recent evidence from the childhood cancer survivor cohort suggests that a range of second cancers including sarcoma, non-melanoma skin and meningioma show strong linear radiation dose–responses for relative risk, while breast cancer shows a linear response with lower increases in relative risk per unit dose, and thyroid cancer relative risk exhibits a saturation and declining induction at doses above 20 Gy [33]. Such observations of tissue-dependent risk patterns and general linearity of the dose–response strongly support the estimates of radiation-associated second cancers following radiotherapy. However, it should be noted that seemingly large relative risks modifying low baseline rates for cancers which are rare in early life are consistent with the observed cumulative incidence of second cancers in cancer survivors [25]. Differences between historical radiotherapy practices and modern treatment modalities will potentially be reflected in future second cancer patterns given the long-term elevation in risk decades after treatment for primary cancer [34].

After controlling for the effect of radiation dose, the relationship between the type of first cancer and the risk of second cancers is much diminished (reviewed in [33]), although there remain some associations which might be explained by combined effects of concurrent chemotherapy or other treatment differences, selection effects, or inherent genetic susceptibility or shared etiology. Research into identifying radiation signatures in radiotherapy-induced cancers [35,36,37] would increase the sensitivity of epidemiology analyses by helping to differentiate between the background of spontaneous second cancers.

## 4. Genetic Variation in Sensitivity to Radiation-Induced Acute and Late Toxicity

The extent to which variability in radiosensitivity is due to intrinsic genetic factors will determine whether patient adverse reactions to radiotherapy, and/or radiotherapy-induced second cancer risk, can be best predicted by classifications based on generic parameters (age, gender, diet, lifestyle factors, etc.) or by individual assessment of genetic profiles. Studies of individuals with known genetic mutations which produce severe phenotypes has firmly established the association between genetics and extreme radiosensitivity. However, the inability to design studies to directly assess acute and long-term radiation injury in normal tissues of healthy volunteers, and the difficultly of long-term radiation-induced cancer incidence studies, complicates determining the heritability of radiation sensitivity in the general population. Instead, most data have been obtained through the use of surrogate measures of normal tissue radiosensitivity, often through ex vivo assays on lymphocytes or fibroblasts.

Since radiation-induced chromosomal damage leading to cell death or loss of cellular reproductive capacity is considered the primary mechanism by which normal tissues suffer injury during radiotherapy, chromosomal aberrations and clonogenic survival represent the classic, most reliable endpoints to predict cellular radiosensitivity [38,39,40]. Clonogenic survival assays usually require cell transformation, which can interfere with the inherent radiosensitivity of cells [41,42,43], and variations in radiation-induced cell survival between different cell types from the same individual complicate assessments [44,45].

The use of two surrogate functional assays of radiation sensitivity in a human twin-pair study calculated a heritability of 59% for radiation-induced apoptosis, and 68% for radiation-induced cell cycle delay [46]. Twin studies showed heritability estimates of 57 to 72% for variations in baseline and radiation-induced micronuclei frequencies depending on the analysis method [47]. An analysis of sensitivity to radiation-induced chromosomal aberrations in a subset of cancer survivors attributed 58 to 78% of the observed variance to genetic factors [48]. Such calculations underlie the general acceptance that genetics accounts for most of the variability in radiation sensitivity (reviewed in [49]).

The contribution of cytogenetics to the understanding of radiosensitivity is well recognized [40,50]. Although no specific chromosomal breakpoint patterns after ex vivo irradiation were observed in a small number of radiosensitive patients [51], in another study, translocations and unstable chromosomal damage were found to be predictive of late tissue toxicity in prostate cancer patients [52,53]. Analysis of spontaneous and irradiation-induced chromosomal aberrations in lymphocytes of healthy donors, cancer patients, individuals with ATM serine/threonine kinase (*ATM*) and Nibrin (*NBS1*) mutations, and radiosensitive patients [54] confirmed that *ATM* and *NBS1* homozygous patients had the highest incidence of aberrations; while among the radiosensitive group, outliers with high chromosomal breakage were detected.

The radiosensitivity of keratinocytes and fibroblasts from radiotherapy patients measured by the clonogenic survival assay correlated with a fraction of unrepairable radiation-induced DNA double-strand breaks measured by the neutral Comet assay [55]. The utility of scoring DNA double-strand break repair foci (γ-H2AX or tumor protein 53 binding protein 1 (53BP1)) as a surrogate measure of cell survival (itself a surrogate for clinical radiosensitivity) has been explored. Pairing γ-H2AX analysis and apoptosis assays in human tumor cell lines was predictive of their radiosensitivity in a standard colony-forming assay [56]. In other settings, the results of a clonogenic survival assay in primary skin fibroblasts derived from pediatric severe combined immunodeficiency (SCID) patients were in concordance with the DNA double-strand break repair efficiency measured by the γ-H2AX foci assay [57,58].

Yet, human fibroblasts lacking *ATM* or tumor protein 53 (P53) showed aberrant DNA repair responses by comet and γ-H2AX assays, but only in the *ATM* mutant lines did these responses correlate with cell survival [59]. Assessing 40 human fibroblast lines representing eight different syndromes with multiple sensitivity assays showed that survival at 2 Gy was inversely proportional to the level of residual DNA double-strand breaks for all genes and assays [60], but no one assay could adequately describe the full spectrum of radiosensitivity. The effects of *ATM*, DNA-activated protein kinase (*DNA-PK*) and ATR serine/threonine kinase (*ATR*) inhibitors on γ-H2AX kinetics in peripheral blood lymphocyte assays confirms that a variety of DNA repair defects have predictable effects on DNA repair kinetics assessed ex vivo [61], and paired γ-H2AX assays and genotyping of candidate single nucleotide polymorphisms has shown evidence for risk alleles in DNA repair genes in healthy individuals [62]. A study in large kindred families demonstrated the heritability of lymphocyte radiation-induced apoptosis responses, with evidence of dominant effects from a single gene locus [63], later reported to be TNF superfamily member 10 (*TRAIL/TNFSF10*) [64]. A genome-wide association study in a large panel of lymphoblastoid lines screened for radiation cytotoxicity also found several polymorphisms and gene expression candidates as candidate biomarkers for the variation in radiation response [65]. The identification of candidate genetic determinants of radiation response which are not canonical radiation-responsive genes (reviewed in [49,66]), suggests that selective approaches that target only obvious candidates may miss the larger pool of genes which collectively define a polygenetic trait such as radiation sensitivity [67,68].

Although the links between genetics and lymphocyte and fibroblast ex vivo responses to radiation are becoming clearer, the translation between such surrogate markers and clinical presentations of radiosensitivity following radiotherapy has proven more difficult (reviewed in [6,69]). In early work, post-irradiation fibroblast cell survival did not correlate with radiotherapy-induced fibrosis [70], while gene expression responses in ex vivo irradiated lymphocytes showed some limited ability to predict radiosensitivity [71]. A set of polymorphisms which were earlier associated with radiation-induced fibrosis were not able to be replicated in later work [72]. A correlation between adverse acute skin reactions and the initial yield and residual number of γ-H2AX foci [73] has been observed in breast cancer radiotherapy patients, while in another study radiotherapy adverse reactions were not able to be predicted by differences in gene expression, apoptosis, residual DNA breaks or chromosomal damage [74]. Other work has shown that both higher initial yields of DNA damage and reduced levels of apoptosis in ex vivo irradiated lymphocytes, postulated to indicate failure to appropriately respond to damage, correlated with increased risk of severe late skin toxicity [75]. Such inverse relationships between lymphocyte apoptosis and chronic toxicity have been observed in other studies [76,77]. Conversely, increased levels of DNA damage and increased lymphocyte apoptosis after ex vivo irradiation have been shown to be correlated to early acute toxicity [78].

## 5. Genetic Variation in Sensitivity to Radiation-Induced Cancer

Since inherited mutations which confer extreme radiosensitivity are frequently associated with both increased spontaneous cancer risk, including syndromic neoplasms, and increased radiation-induced cancer risk [24,31], the question arises as to whether the general spectrum of radiosensitivity (for acute and late effects of radiotherapy) is mirrored in variations in susceptibility to radiotherapy-induced second cancer. The mechanisms underlying enhanced radiotoxicity in normal tissues, such as increased cell death, inflammation and impaired tissue regeneration, are also implicated in carcinogenesis. Although, as opposing relationships between radiation-induced apoptosis and radiotherapy-induced adverse effects show, it is not a simple case that any given genotype will equally affect both pathways. The data available to characterize the link between genotypes and cancer susceptibility largely follow the same approaches as for the radiotoxicity studies discussed above [79], with both epidemiology studies and the use of functional assays used as surrogate markers of cancer sensitivity (reviewed in [80]). These studies can involve the molecular characterization of cancers arising in radiation-exposed populations to investigate inter-individual variations in sensitivity ([81]; reviewed in [82]), radiosensitivity testing using functional assays in families suspected of heightened cancer predisposition [83], the identification of sub-populations at increased risk of second cancer after radiation exposure [84,85], or the specific investigation of radiation-induced cancer in individuals with known genetic predispositions [86,87].

Perhaps the most widely studied association is between familial breast cancer mediated by DNA repair associated *BRCA1* and *BRCA2* mutations and radiation-induced cancer/second cancer [88,89,90,91]. Investigations into the association between single nucleotide polymorphisms and the development of radiation-induced second malignancies in children treated for Hodgkin’s lymphoma have identified several interesting candidates [92], however, similar results were not observed for survivors of adult-onset Hodgkin’s lymphoma [93].

The ability to use functional assays to identify individuals who might be susceptible to spontaneous and/or radiation-induced cancer, but without known family history or genetic pre-disposition, would be a key tool in cancer prevention. Lymphocytes from a subset of breast cancer patients and their first-degree relatives showed sensitivity to radiation-induced micronuclei [94], while another study of radiation-induced chromosomal aberrations and apoptosis found no overall difference in the responses of breast cancer patients and their matched controls, but with a possible effect restricted to patients with strong family history of breast cancer [95]. Unaffected parents of retinoblastoma patients exhibited cellular radiosensitivity despite not carrying the causative mutant allele, postulated to be consistent with a predisposition to generate de novo mutations [96]. Similar levels of sensitivity, equivalent to those seen in *ATM* heterozygotes, could also be found in fibroblasts cultured from a subset of otherwise normal donors.

## 6. Selection of a Functional Assay

It seems likely that comprehensive genetic analysis will eventually provide the best predictive power for radiation sensitivity, both for acute and late toxicity and susceptibility to radiation-induced cancer. However, the current labor and financial costs involved make routine use of whole-genome approaches prohibitive, and our ability to interpret a myriad of genetic variations between individuals into a reliable predictor of radiosensitivity is limited once single-locus, high-penetrance traits are excluded. Currently, genetic and protein analysis of patient cells is able to identify mutations/variants that are likely to result in radiosensitivity, but this requires investigation on a sample-by-sample basis and has no formal guidelines for the interpretation of results. As time- and cost-constraints improve with time, and an ever-growing knowledge base provides data for algorithms to accurately assess the impact of a complex genotype/proteomic signature, such radiogenomic investigations are invaluable alongside functional assessments of radiosensitivity.

While facing their own limitations, the application of functional assays does seem to provide a firm basis to assess individual radiation responses. A perfect predictive assay should be minimally invasive; easily established; sensitive; provide a clear result in a timely fashion so the outcome can be incorporated into clinical decision-making; amenable to automation; and accurate, with a low probability of falsely predicting abnormal sensitivity [97]. Of the variety of functional assays utilized in investigations of radiosensitivity, many have failed to reach clinical applicability, largely due to limitations in sensitivity, reproducibility, reliability, and delayed availability of results, incompatible with the urgency of pre-radiotherapy planning.

Functional assays in peripheral blood lymphocytes based on radiation-induced γ-H2AX kinetics [98,99] have shown promise in the prediction of radiosensitivity [100]. The general applicability of the method relies on the fact that phosphorylation of the histone variant H2A.X (forming γ-H2AX) is one of the earliest events in the DNA damage response, and facilitates DNA repair [101]. The amplification of the γ-H2AX response along the mega-base region surrounding DNA double-strand breaks allows a single break site to be detected by a focus of modified histones which can be visualized by microscopy. The radiation dose-dependent kinetics of foci induction and repair is rapid and follows a common pattern in all normal tissues, with residual foci that persist more than 24 hours after irradiation exposure representing incompletely repaired DNA double-strand breaks [100]. One caveat of the γ-H2AX assay is that it cannot distinguish between faithful DNA repair and misrepair, nor between radiation-induced simple and complex DNA damage [102]. The presence of radiation-induced complex DNA damage (which is rarely encountered from endogenous damage) can lead to genomic instability and the triggering of defensive signaling [103,104] that can activate tissue-level responses, such chronic inflammation. In parallel, genetic mutations in DNA repair genes that can underlie radiosensitivity (as detected by the γ-H2AX assay) can also contribute to radiation-induced cancer through such systemic mechanisms [105].

The rate of γ-H2AX foci repair slows down with age [106,107,108], suggesting that the assay can detect both inherent and dynamic sensitivity to persistent radiation damage. The assay has successfully identified patients with known mutations in *ATM*, *NBS1*, fanconi anemia complementation group A (*FANCA*), DNA ligase 4 (*LIG4*) and others (reviewed in [99]). Patterns of γ-H2AX kinetics corresponding to severe or mild mutations in core non-homologous end joining (NHEJ) factors have also been identified [57,58,109,110], and the sensitivity of the assay allows detection of minor alterations such as *ATM* and DNA cross-link repair 1C (*DCLRE1C*) including heterozygotes [58,111].

A large series of studies have aimed to demonstrate correlations between post-irradiation γ-H2AX responses and acute and late radiosensitivity, or radiation-induced cancer risk, across a large variety of cancer patient cohorts [112,113,114,115,116,117,118,119,120,121,122,123,124,125,126,127,128], with mixed results. Out of 22 relevant studies published in 2008–2016, γ-H2AX analysis based on the comparison of over-responders, non-over-responders and normal blood donors, thirteen studies demonstrated an ability of the assay to predict radiosensitivity-associated normal tissue toxicity whereas nine studies showed that γ-H2AX is an unreliable predictive marker [129]. Variation in the methods used across all the studies suggests that the assay design (including the choice, storage and culture of cells) and the parameters chosen to act as a quantitative metric influence the utility and reliability of the γ-H2AX assay. Standardization of techniques and unbiased analysis protocols may help to overcome some of the challenges associated with use of a predictive assay in the clinic.

Our group employed an assay of the kinetics of ex vivo radiation-induced γ-H2AX foci and co-localization of these foci with 53BP1, to compare the cellular radiosensitivity in peripheral blood lymphocytes of former radiotherapy patients who developed abnormally severe late radiation toxicity with corresponding control patients matched for age, gender, tumor type, radiation dose, and radiotherapy duration [129]. The results were validated in an additional tissue type, eyebrow hair follicles. We found that the combination of the fraction of the unrepairable component and repair rate derived from non-linear regression analysis of foci repair kinetics was the most powerful predictor to distinguish between radiosensitive and non-radiosensitive patients, with a 97% predictive power. A small-scale genetic analysis from these patients showed that variations in DNA repair genes could be found in a subset of patients, while others showed functional radiosensitivity as assessed by γ-H2AX kinetics, despite no detected DNA repair mutations. The ability for γ-H2AX analyses to be conducted rapidly, to be automated (both through imaging and analysis technology, and non-image based techniques), and to require only minimally-invasive blood sampling, means that despite the challenges involved in optimizing the assay for clinical prognostication, it remains one of the key leads in radiosensitivity functional assays [100,130].

A competing assay which represents the leading alternative to the γ-H2AX assay is the assessment of radiation-induced apoptosis in normal lymphocytes. The assay circumvents the extensive time required for clonogenic survival assays by measuring short-term cell death in irradiated primary cells. Recent results have confirmed the predictive power of the assay for both late toxicity and tumor hypersensitivity [131], with evidence that the response of particular lymphocyte subsets may give even stronger correlations. This has implications for the γ-H2AX assay which may also benefit from restriction to particular cell subsets. The apoptosis assay has the benefits of fast turnaround and the possibility of automation; however, unlike DNA repair kinetics which can provide insight into the mechanism of repair deficiency through time- and dose-responses [129], it produces only a single value on which to set a decision threshold. It also is likely to correlate better with the risks of acute and late toxicity than with second cancer risk, given the opposition between cell survival and cell death for carcinogenesis.

## 7. Opportunities for Risk Mitigation in Sensitive Patients

There are only limited data available on the long-term outcomes of radiotherapy in individuals harboring a known cancer susceptibility, such as in Li–Fraumeni syndrome patients treated for first breast cancer [87]. If cancer patients with an increased sensitivity to radiation-induced second cancer could be identified before radiotherapy, through either functional assay or genomic profiling, the challenge becomes: what strategies could be employed to provide successful primary cancer treatment while minimizing the risk of a treatment-related malignancy? An obvious approach is to reduce the radiation dose delivered to at-risk tissues. The incorporation of on-beam time as an optimizing parameter into treatment planning systems (as a surrogate for minimizing leakage doses) for IMRT is such an example of a generic strategy; although, how such considerations are weighted within the algorithms ultimately depends on clinical decision-making. Whilst the general reduction of radiation dose outside the target volume is already a cornerstone of advances in radiotherapy technology (reviewed in [132]), it is possible that understanding the specific sensitivities of a given patient might mean a radiotherapy plan that avoids a uniquely sensitive tissue at the expense of other normal tissues might be preferred over a generic avoidance of all tissues. These decisions will be informed by our understanding of the tissue-specific toxicities which present the greatest limitation to tumor dose-escalation and the greatest impacts on quality of life (such as bladder, rectal and erectile dysfunction in prostate cancer radiotherapy), as well as the organs at greatest risk of radiation-induced cancer within the relevant treatment field. Alternatively, the balance between protecting one tissue from acute toxicity and protecting another from increased risk of radiation-induced cancer might be informed by knowledge of the risks unique to an individual sensitive patient. However, the evidence suggests that the organs and tissues at most risk of radiation-induced second cancer [133] are similar to those susceptible to acute and late toxicity (such that both would be protected by the same plan, [134]). Further, while normal tissue toxicity can be avoided altogether by remaining below the effective threshold dose, second cancer risk generally scales linearly with dose at the levels relevant for radiotherapy exposures to normal tissue [33], such that purposely risking normal tissue toxicity in one tissue in order to provide only a proportional change in cancer risk in another organ is not a palatable proposition. Likewise, reducing tumor dose to protect normal tissues involves a delicate balance between the risk of short-term treatment failure and the long-term risk of future cancer.

The simplest option is to avoid radiotherapy altogether in radiosensitive individuals. However, many of the epidemiological studies which support radiotherapy-associated second cancer also implicate chemotherapy as a strong risk factor [135], and many of the genetic variations that underlie radiosensitivity may also confer equal or greater sensitivity to cytotoxic agents [33]. This may leave surgery as the only option, but one which may not be possible in many cases of unresectable or inoperable tumors. A parallel problem arises in radiosensitive individuals identified only after exposure to radiation, such as through potential triage following a radiation emergency using function-assay screening [136]. The combination of both estimated exposure through bio-dosimetry and the identification of at-risk individuals may allow the classification of not only those amenable to countermeasures to reduce acute radiation sickness, but also those exposed to lower doses but who might benefit from enhanced cancer surveillance measures. This also raises the complication of radiosensitivity interfering with bio-dosimetry assays, depending on which metrics are used.

We have reviewed other potential avenues which might reduce the risk of second cancers without compromising the efficacy of radiotherapy tumor control in [137]. Such approaches include the systemic delivery of radioprotective agents which can selectively protect normal tissues without desensitizing tumor cells, topical or localized delivery of non-selective radioprotective agents, and the manipulation of systemic effects which could act to increase resistance to cancer induction/progression on a larger scale (such as through immune surveillance, abscopal effects or other physiological mechanisms). A final opportunity is in the domain of tumor radiosensitivity, which might provide the chance to increase tumor control in patients with inherently radioresistant or radiosensitive cancers by exploiting an individualized treatment approach [138].

## 8. Conclusions

While there are clear commonalities between radiosensitivity as it pertains to acute and late toxic reactions after radiotherapy, and genetic predisposition to radiation-induced cancer, the two concepts cannot be considered as equivalent. While cell death underpins much of the manifestation of normal tissue toxicity, the death of cells with DNA damage represents a fundamental cancer protection mechanism. Ultimately, the exact pathway which mediates the altered balance between DNA damage recognition, repair and the choice of the cell to survive or die, will determine whether a genetic trait that leads to radiosensitivity also alters the risk of radiation-induced cancer. Ultimately, better understanding of why a given patient exhibits cellular radiosensitivity, either through genetic investigation and analysis, or through complimentary functional assays which define the mechanism of sensitivity are needed to accurately refine risk estimates on an individual basis. This can ultimately help to improve radiotherapy planning and practice, and assist patients to make informed choices about their care.
